# Derivatization of Methylglyoxal for LC-ESI-MS Analysis—Stability and Relative Sensitivity of Different Derivatives

**DOI:** 10.3390/molecules23112994

**Published:** 2018-11-16

**Authors:** Stefan Fritzsche, Susan Billig, Robby Rynek, Ramarao Abburi, Elena Tarakhovskaya, Olga Leuner, Andrej Frolov, Claudia Birkemeyer

**Affiliations:** 1 Institute of Analytical Chemistry, Faculty of Chemistry and Mineralogy, University of Leipzig, 04103 Leipzig, Germany; stefan.fritzsche@uni-leipzig.de (S.F.); billig@uni-leipzig.de (S.B.); r.rynek@live.de (R.R.); rams.abburi@gmail.com (R.A.); elena.tarakhovskaya@gmail.com (E.T.); leuner@ftz.czu.cz (O.L.); 2 Institute of Pharmacy, Faculty of Medicine, University of Leipzig, 04103 Leipzig, Germany; 3 Department of Chemistry, Krishna University, Machilipatnam 521001, Andhra Pradesh, India; 4 Department of Plant Physiology and Biochemistry, Faculty of Biology, Saint Petersburg State University, Saint Petersburg 199034, Russia; 5 Library of the Russian Academy of Sciences, Saint Petersburg 199034, Russia; 6 Faculty of Tropical AgriSciences, Czech University of Life Sciences Prague, 165 00 Prague-Suchdol, Czech Republic; 7 Department of Bioorganic Chemistry, Leibniz Institute of Plant Biochemistry, 06120 Halle (Saale), Germany; andrej.frolov@ipb-halle.de

**Keywords:** carbonyl derivatization, phenylhydrazine, phenylenediamine, hydroxylamine, water analysis, lipoxidation

## Abstract

The great research interest in the quantification of reactive carbonyl compounds (RCCs), such as methylglyoxal (MGO) in biological and environmental samples, is reflected by the fact that several publications have described specific strategies to perform this task. Thus, many reagents have also been reported for the derivatization of RCCs to effectively detect and quantify the resulting compounds using sensitive techniques such as liquid chromatography coupled with mass spectrometry (LC-MS). However, the choice of the derivatization protocol is not always clear, and a comparative evaluation is not feasible because detection limits from separate reports and determined with different instruments are hardly comparable. Consequently, for a systematic comparison, we tested 21 agents in one experimental setup for derivatization of RCCs prior to LC-MS analysis. This consisted of seven commonly employed reagents and 14 similar reagents, three of which were designed and synthesized by us. All reagents were probed for analytical responsiveness of the derivatives and stability of the reaction mixtures. The results showed that derivatives of 4-methoxyphenylenediamine and 3-methoxyphenylhydrazine—reported here for the first time for derivatization of RCCs—provided a particularly high responsiveness with ESI-MS detection. We applied the protocol to investigate MGO contamination of laboratory water and show successful quantification in a lipoxidation experiment. In summary, our results provide valuable information for scientists in establishing accurate analysis of RCCs.

## 1. Introduction

The research interest in the role of reactive carbonyl compounds (RCCs) for environmental and biomedical aspects has grown tremendously ever since their relevance for human metabolism was first discovered [1,2,3]. One of the biologically most important representatives of this compound group is methylglyoxal (MGO), which has been suggested to be involved in many lifestyle diseases, such as diabetes [4,5], cancer [6], aging [7,8] or even behavioral phenotypes, such as anxiety [9] and depression [10]. In biological samples, reactive carbonyl compounds are usually formed from carbohydrate and lipid metabolism [11,12], while in the environment, they may originate from degradation of volatile organic substances in the atmosphere, such as isoprene [13]. However, the analysis of RCCs is very challenging as most of them are UV-inactive and as they often feature only intermediate polarity. In addition, small RCCs are volatile, which complicates sample handling. Due to the transient, reactive nature of the analytes (e.g., MGO in the atmosphere has an average lifetime of 1.6 h [13]), trap reagents are often employed to avoid further reaction of the target compounds with other sample components. These trap reagents are usually selected to obtain derivatives with enhanced sensitivity compared to the parent compound with respect to the anticipated analytical detection technique.

**Figure 1:**
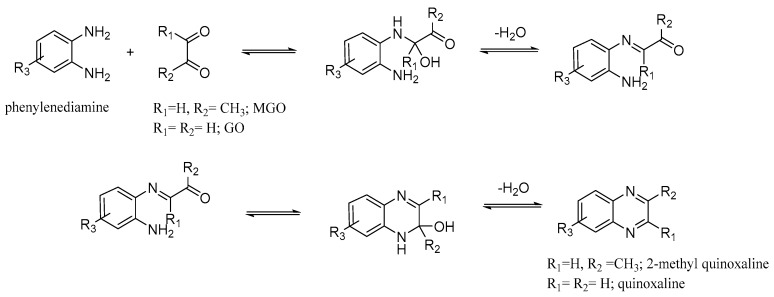
Reaction of dicarbonyl compounds (here methylglyoxal) with phenylenediamines (here *o*-phenylenediamine) as starting material. After addition of the two compounds, water is eliminated to form the mono-imine (**top**), which in a second addition-elimination cycle forms the quinoxaline (**bottom**).

Consequently, there are a few requirements for an optimal derivatization (or trap) agent: (i) solubility in or efficient miscibility with the sample or sample extract; (ii) a very fast reaction with the target compound (for efficient trapping of the analyte); (iii) modification of the original RCC to a derivative that can be very sensitively detected by the chosen analytical technique; and (iv) sufficient stability of the derivatives during analysis [14]. As RCCs are particularly reactive against amino groups, they are most often derivatized with reagents featuring a free amino group, such as hydroxylamines [15,16], amines [17,18], or hydrazines [14,19,20,21]. The reaction proceeds via the very well-known addition/elimination mechanism. This is depicted in Figure 1 with α,β-dicarbonyl compounds and phenylenediamines as an example.

For analysis of small RCCs, *O*-(2,3,4,5,6-pentafluorobenzyl)hydroxylamine (PFBHA, [16]) is one of the most frequently applied reagents for subsequent analysis with gas chromatography (GC) coupled with electron impact ionization mass spectrometry (EI-MS) [22,23]. Apart from stability, their derivatives feature a decreased volatility compared to the original compound and a lower polarity due to the shielding of the original molecule by the large nonpolar substituent. In addition, a very abundant, diagnostic fragment (*m/z* 181) enables very sensitive quantification of the derivatives in EI-MS. However, in aqueous samples, PFBHA derivatives first need to be extracted into a GC/MS-suitable organic solvent by liquid/liquid (l/l) partitioning to lengthen the sample preparation procedure and possibly decrease the recovery while increasing the analytical variance. Moreover, due to the required volatility of the analyte, the application of multitargeted analysis of RCCs by GC is limited to RCCs with small molecular weight, and two-step derivatization protocols need to be employed for hydroxylated representatives and carboxylates [24,25].

In contrast, liquid chromatography (LC) coupled with electrospray ionization (ESI) MS allows the direct analysis of aqueous samples, rendering l/l extraction of the derivatives from biological samples unnecessary. For LC-MS analysis of target analytes containing aldehyde and/or keto group, reaction with amines—such as *o*-phenylenediamine derivatives with RCCs resulting in imines or quinoxaline derivatives with α,β-dicarbonyl compounds and phenylenediamines (PD)—is frequently used (Figure 1). For hydrazines, the corresponding hydrazones are formed, while oximes are formed for hydroxylamines [16,20,24,25,26,27]. The introduction of small aromatic residues improves the required desorption of the target analyte ion during the ionization process. In addition, any other substituent of the reagent apart from the required amino group can be selected to further enhance the reactivity of the reagent (e.g., electron-donating substituents enhance nucleophilic attack during the addition) or improve ionization efficiency during electrospray. These are two very important requirements for successful analysis (electron-donating substituents also enhance the basicity of a compound for determining characteristic of a good ESI sensitivity [28]).

**Figure 2:**
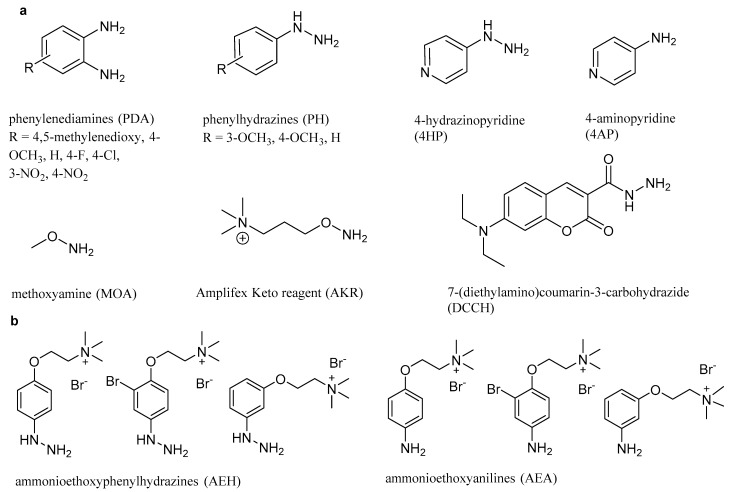
Chemical structure of the reagents tested for derivatization of methylglyoxal with subsequent ESI-MS detection: (**a**) commercially available compounds; (**b**) synthesized reagents for derivatization of carbonyl compounds, from left to right, 2-(4-hydrazineylphenoxy)-*N*,*N*,*N*-trimethylethan-1-aminium bromide (4-AEH), 2-(2-bromo-4-hydrazineylphenoxy)-*N*,*N*,*N*-trimethylethan-1-aminium bromide (3-BrAEH), 2-(3-hydrazineylphenoxy)-*N*,*N*,*N*-trimethylethan-1-aminium bromide (3-AEH), and the corresponding anilines including 2-(4-aminophenoxy)-*N*,*N*,*N*-trimethylethan-1-aminium bromide (4-AEA) according to Eggink et al. [24].

Many reagents and optimized protocols for derivatization of RCCs prior to analysis with LC-MS have already been reported. One of the most well-known reagents is 2,4-dinitrophenylhydrazine (DNPH), which was originally chosen for sensitive detection of RCCs with UV [20]. However, in the recent past, many alternatives to this reagent have been reported to provide a more sensitive and selective, and thereby more accurate, analysis. Moreover, new reagents have been tailored for use with mass spectrometric detection [24] to achieve a higher analytical sensitivity with this particular technique. In this report, we present a survey of 21 compounds for derivatization of MGO prior to LC-ESI-MS analysis with respect to analytical efficiency and robustness (stability). This consisted of 15 commercially available compounds (eight phenylenediamines, four hydrazines, two hydroxylamines and a coumarin hydrazide, Figure 2a) and six synthesized compounds (three hydrazines and their corresponding anilines featuring a permanent charge, Figure 2b).

In this paper, we present a thorough comparison of the reagents to identify related problems and illustrate major obstacles in the accurate analysis of RCCs, which is still a challenge [29]. In conclusion, we discuss particularly efficient reagents and the requirements for further optimization and finally present the successful application of the best performing protocol for quantification of methylglyoxal in a lipoxidation experiment.

## 2. Results and Discussion

### 2.1. Relative Response of Methylglyoxal Derivatives in LC-MS Analysis

**Figure 3:**
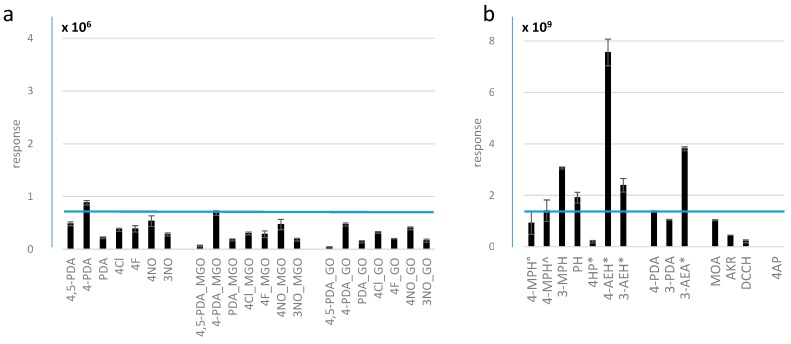
Relative abundances of the different reagents and their corresponding derivatives. Response is normalized to the peak of the two co-eluting products of MGO with 4-methoxyphenylenediamine within each experiment, the isomers 6- and 7-methoxy-2-methylquinoxaline (4-PDA_MGO in Figure 3a and 4-PDA in Figure 3b), illustrated by the blue horizontal line. (**a**) Relative response of the protonated molecular ions of phenylenediamine reagents (left) compared to the quinoxaline reaction products after incubation with MGO (middle) and GO (right). (**b**) Relative response of the most abundant reaction product of methylglyoxal with the corresponding reagent, including phenylhydrazines, methoxyphenylenediamines, and hydroxylamines. (° indole after two days incubation, ^ diimine after four days incubation, * monoderivative). The structure of all reagents is presented in Figure 2. All abbreviations are listed in Supplementary Table S2.

Although theoretically indicative, the ESI-MS responsiveness of carbonyl derivatives can be quite different from that of the original amine reagent; therefore, the responsiveness of the reagent is only a rough indicator for predicting the relative response of the corresponding derivative. For example, Figure 3a compares the change in response pattern of the protonated molecular ion of the phenylenediamines (left) and either their methylglyoxal (middle) or glyoxal (GO, right) quinoxaline derivatives depending on the phenylenediamine substituent R_3_ (see Figure 2a). The corresponding *m/z* values of the MGO derivatization products that were used for quantification are given in Supplementary Table S1.

Among the phenylenediamines, signal intensity of the 4-methoxyphenylenediamine (4-PDA) product was consistently higher compared with those of other phenylenediamines, which was confirmed for different molar ratios of reagent and aldehyde (data not shown). As expected, the derivatives of reagents with strong mesomeric effect exhibited the highest signals with +M (e.g., methoxy) being better than -M (e.g., NO, nitro), and *para*-substituted (4-position) better than *meta*-substituted ones (3-position). According to these results, 4-PDA would be the best phenylenediamine reagent for detection of low amounts of methylglyoxal. Apart from ionization efficiency, the relative response of the reaction products may be caused by different reaction yields, e.g., due to insufficient incubation time. Thus, after 3 h incubation time, only 50% reaction yield was reported when using 2,4-dinitrophenylhydrazine (DNPH) for MGO derivatization [30], while reactions with 4-PDA and 4,5-methylenedioxyphenylenediamine (4,5-PDA) have been reported to be nearly complete after just 1 h [26,31]. These findings were in agreement with our results. Consequently, the characteristics supporting ionization efficiency, e.g., electron-donating substituents enhancing the basicity of compounds as the most important criterion for ESI-MS responsiveness of nitrogen bases [28], often foster the reactivity of the compound as well, resulting in faster reaction times. (Note that DNPH, which is the most commonly employed phenylhydrazine, was not included because with mass spectrometry the derivatives are usually detected in ESI negative ion mode, meaning the response cannot be easily compared to the other reagents. Moreover, the slow reaction times contradict the purpose of our study, which was to find a reagent candidate with a fast reaction time). However, we did not notice a significant time-dependent response effect between 2 and 4 h incubation time for all of the commercially available reagents and no response enhancement for the derivatives after that time, which also generally resulted in a satisfactory standard deviation of the replicates. As a general rule, signal response of the derivatives from reagents with electron-donating substituents slowly decreased up to 50% during the course of a day, so we prepared all samples for response comparison in situ.

4-PDA has only recently been introduced as a derivatization agent for methylglyoxal [26]. Among the tested, commercially available phenylenediamines, this reagent seems to produce the most sensitive signal response. Thus, we compared this particular reagent to phenylhydrazines, another group of commonly employed reagents for analysis of carbonyls that also promises to provide excellent performance for derivatization. The results are illustrated in Figure 3b.

It is clear that 3-methoxyphenylhydrazine (3-MPH)—which to our knowledge has not been suggested yet for derivatization of methylglyoxal—outperformed all other commercially available reagents we tested in our experiments. Only our synthesized phenylhydrazine carrying a permanent charge in *para* position (4-AEH) provided a higher intensity, although it mostly reacted with just the aldehyde function and we observed mainly monomers. For comparison, Table 1 lists the corresponding estimated limits of detection (LOD) for selected protocols, i.e., using the reagents 4-PDA, 3-MPH, 3-ammonioethoxyphenylhydrazine (3-AEH), 3-ammonioethoxyaniline (3-AEA), coumarin carbohydrazide (DCCH), Amplifex™ Keto Reagent (AKR, ABSciex, Framingham, MA, USA), and 4-MPH.

The pattern of LODs did not exactly follow the relative response as obtained from signal intensities at 50 µM MGO (0.5 nmol injected). Thus, for 3-AEA, a surprisingly high value was found, while 4-PDA had a surprisingly low LOD in contrast. Our comparison confirms the notion that assessment of relative sensitivities based on peak areas far above the detection limit does not necessarily help to find the most sensitive protocol. According to these results, 4-PDA might be the preferred reagent by far for analysis of α-oxo-aldehydes, which quinoxaline derivative(s) of methylglyoxal (and glyoxal, not shown) were detected particularly sensitive. However, at higher concentrations of monocarbonyl compounds, multiple derivatives could be produced from phenylenediamines, which can be avoided when using the second-best candidate reagent, i.e., 3-MPH. Furthermore, the production of a diagnostic daughter ion from 3-MPH derivatives, *m/z* 122, provides a great advantage for identification of unknown carbonyl compounds by MS/MS analyses.

A drawback of using phenylhydrazines for quantification of nonsymmetric dicarbonyl compounds, such as methylglyoxal, compared to phenylenediamines is the formation of two mono- and two main bis-derivatives (for the latter, four isomers would be possible). For 3-MPH, the less abundant bis-derivative was present as a shoulder peak under our conditions, so we quantified the signal as the more robust sum of both peak areas. The same applied to the coumarin carbohydrazide DCCH, which was also the only reagent considerably retained beyond the dead volume of the LC column. Here, the two MGO monoderivatives eluting within the broad tailing of the reagent peak were nearly twice as abundant as the late eluting bis-derivatives, indicating insufficient reactivity against ketones. (Note that another reason for the observed low abundance of the bis-derivative might be the expected very poor solubility, with a logS of 6.78 in the ChemAxon’s solubility predictor [34] corresponding to maximal 3 µM dissolved substance vs. 50 µM starting material used in this experiment.). Considering the use of the reagent for a generally applicable multimethod to analyze aldehydes and ketones beyond the analysis of α-oxo-aldehydes, 3-MPH would still be favorable due to its nonselective high reactivity. In return, the presence of two isomers also enables an easier identification of corresponding derivatives in an unknown mixture or for derivatization of unknown carbonyl compounds.

**Figure 4:**
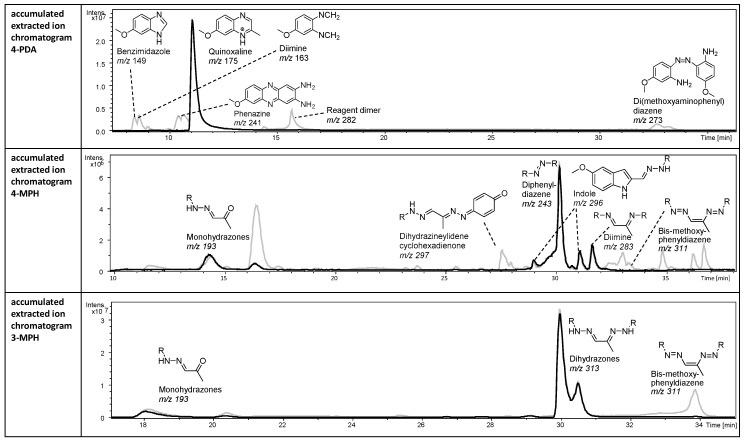
LC-ESI-MS analysis of reaction mixtures of methylglyoxal with phenylenediamines or phenylhydrazines. **Top**: reaction mixture with 4-PDA. **Middle**: reaction mixture with 4-MPH. **Bottom**: reaction mixture with 3-MPH. Black: ion current of selective mass traces, *m/z* 175 (extracted ion chromatogram for the reaction mixture with 4-PDA) or *m/z* 193, 243, 283, 296, 313 (reconstructed ion current for the reaction mixtures with 4-MPH and 3-MPH). Grey: base peak chromatogram, BPC (intensity of the base peak at a given retention time).

The most curious result, however, is the bad performance of 4-MPH as derivatizing reagent. With this reagent, the base peak chromatogram of the reaction mixture was dominated by multiple degradation products rather than by the expected MGO derivatives. Thus, the only intermediate response of the main derivative (*m/z* 296) and the corresponding high variance of this species may be the consequence of ion suppression by a very abundant coeluting reagent dimer (*m/z* 243, see Section 2.2.1 or Figure 4). Stability tests suggested that extensive oxidation processes within the reaction mixture hampered reproducibility of the results and led not only to the degradation of derivatives and reagent but also to precipitations in reaction mixtures with high concentration of GO.

### 2.2. Extent of Degradation, Solubility, and Practical Considerations

#### 2.2.1. Multiple Reaction Products in the Chromatograms of 4-MPH and 4-PDA Reaction Mixtures

Particularly with the successful derivatization reagents, we noticed extensive, time-dependent reactive processes within the reaction mixtures, which were not inhibited by addition of antioxidants such as 2-mercaptoethanol or di-tert-butyl-hydroxytoluene (BHT) after incubation (not shown); these results were in agreement to the observations reported by Nemet et al. [31]. Indeed, the addition of mercaptoethanol, which has been described for the derivatization with 4-PDA [26], led to a slightly but consistently reduced response (90%) of the MGO derivative that was even more prominent for the phenylhydrazines. Therefore, we omitted this additive from our comparison. However, after the addition of mercaptoethanol, the response of the reagent dimer peaks, *m/z* 241 and 273, indeed decreased, indicating an inhibition of reagent polymerization. Moreover, a reduced form of the reagent (e.g., *m/z* 137 for 4-PDA) was observed in mercaptoethanol-containing preparations. 

Figure 4 illustrates the appearance of typical LC-MS chromatograms obtained from the reaction mixtures of MGO, with 4-PDA, 4-MPH, and 3-MPH as an example to illustrate the formation of such by-products.

In the 4-PDA reaction mixture, *m/z* 175 from the protonated reaction product methoxy-2-methylquinoxaline exhibited the most abundant signal. Other abundant peaks with *m/z* such as 149, 163, 241, 282, and 273 have been earlier shown to appear after electrolytic oxidation of the reagent during the electrospray ionization process [35]. As we found the compounds corresponding to these peaks also separated in LC-MS analysis of the reaction mixture, we concluded them to have been formed spontaneously here by reagent oxidation in solution. In addition, after multiple measurements, we observed a very abundant compound with *m/z* 124 eluting at higher acetonitrile (ACN) phase. This *m/z* corresponds to another potential degradation product of 4-PDA—4-methoxyaniline; the signal hardly decreased even after long flushing times of the column. In contrast, the signal intensity of the MGO derivative methoxy-2-methylquinoxaline itself was reduced to 75% after 12 h.

The different hydrazines are known to produce many analytical artifacts in solution, such as by degradation to the corresponding aniline, aryldiazene, or diarylamine [36,37]. Nevertheless, they have long been used as derivatization agents of carbonyls for analytical purposes [20]. This is most likely due to the good sensitivity that can be achieved, the sufficient stability of the target derivatives, and reproducibility [38]. The stability of hydrazines and their derivatives from the reaction with carbonyl compounds, i.e., the hydrazones, is related to the type of substituent and the extent of protonation of the hydrazine/hydrazone group. Thus, phenylhydrazines with electron-donating substituents, such as the alkoxy phenylhydrazines used in this study, are less stable but are much more reactive at the same time due to the higher electron density in the aromatic ring and at the hydrazine group itself. Last but not least, electron-donating substituents make their carrier molecules particularly responsive to ESI-MS in positive mode [28] and promise to introduce high sensitivity to the analysis of the derivatives, a point that is particularly relevant for our investigation.

At low pH, these electron-rich phenylhydrazines exist as the more stable ions; however, degradation to the corresponding aniline already starts in these conditions [39]. Thus, we tentatively identified the diimine with 4-MPH (*m/z* 283, Figure 3) and mixed aniline/hydrazine derivatives with our synthesized, permanently charged phenylhydrazines (data not shown). Moreover, fast reagent polymerization was indicated by the presence of dimethoxyphenyldiazenes from 4-MPH and, to a lesser extent, 3-MPH (*m/z* 243) (Note that reagent dimers were also immediately observed for the phenylenediamines, e.g., *m/z* 241 and 273 for 4-PDA in Figure 4). In agreement, in the reaction mixture of 4-MPH with high concentrations of GO in 20% ACN, a precipitation already occurred after 2 h (Note that no precipitates were observed for MGO at used concentrations).

In the analysis of the reaction mixture from the derivatization with 4-MPH, only a small signal of a tentative oxidation product of the expected derivative—the dehydro-dihydrazone, i.e., the propenediyl-bis(methoxyphenyl)diazene (*m/z* 311)—was detected instead of the dihydrazone, while the main peak appeared to be the di(methoxyphenyl)diazene (*m/z* 243), a chemically formed reagent dimer whose formation should be particularly favored with the methoxy group in *para* position to the hydrazino group. The two main products with MGO were the tentatively identified diimine (*m/z* 283) and an indole (*m/z* 296) related to the MGO–dihydrazone intermediate, which formally cleaves ammonia in a [3,3]-sigmatropic rearrangement known as Fischer indole synthesis. In summary, a minimum of eight abundant, reagent-borne species and six derivatives of MGO were detected in the reaction mixture. The reaction speed of all these products was found to be inversely related to the organic content in the solvent composition of the reaction mixture (data not shown). Consequently, 4-MPH was deemed not suitable for derivatization in LC-MS profiling of reactive carbonyl compounds, and the protocol would require further method development before efficient application. However, we refrained from further optimization given the only-moderate responsiveness for MGO with this protocol.

#### 2.2.2. 3-MPH, Anilines, and Hydroxylamines Exhibit Higher Stability Compared to 4-MPH

In comparison to 4-MPH, 3-MPH produced considerably less by-products (Figure 4); not only were the tentative diphenyldiazene, diimine, and demethylated dihydrazone absent from the reagent mixture, but the monohydrazones also had a very low relative intensity. Thus, 3-MPH produced the expected dihydrazone derivative as base peak in the corresponding chromatogram (*m/z* 313). However, the bis-diazene was also observed due to oxidation in the reaction mixture. In agreement, the derivative of our 3-substituted aniline also gave higher signals compared to the 4-subsituted analogue (data not shown). However, the stability of the 3-MPH derivative was still not satisfactory; after 2–3 hours, signal intensity decreased constantly to 50% at 12 h. However, as response with this reagent was favorable compared to other reagents, we tested the influence of other parameters such as reaction buffers at different pH, the addition of antioxidants or the reducing agent NaCNBH_3_, and storage at lower temperature. Finally, we observed that in higher organic phase, the stability of the derivatives was crucially enhanced, which led to the development of an optimized protocol; shortly after mixing (after ~30 min) the reagent and target analyte, the aqueous sample was almost completely dried and needed to be re-dissolved in methanol before analysis. These samples did not produce any signal decline over the tested period of 24 h, providing satisfactory stability for proper handling in the lab. Moreover, the procedure offers the possibility of sample concentration with respect to a reasonable evaporation time below 2 h in the vacuum centrifuge (otherwise lyophilization might be recommended). The estimated LOD did not change in comparison to immediate analysis in the aqueous sample.

For 3- and 4-PDA using the intensity of the reagent dimer as an indicator, polymerization [35] was observed with both reagents from the very beginning, i.e., after 2 h incubation. The intensity of the signal corresponding to 5-methoxy-3-methylquinoxaline, the product with 3-PDA, was only 75% of the corresponding product from 4-PDA (Figure 3b). However, in agreement with the extent of degradation of the *meta*- compared to the *para*-substituted phenylhydrazine, the intensity of the corresponding reagent dimer peak was 10 times higher in 4-PDA compared to 3-PDA. 

Unlike the phenylenediamines and phenylhydrazines, the anilines synthesized according to Eggink et al. [24] appeared to be rather stable. In addition, the MGO derivatives of the anilines provided an excellent stability within one week storage. Given their competitive signal response (Figure 3b), reagents of this type would have been the most promising approach for derivatization of aldehydes to achieve a very sensitive ESI-MS detection within our comparison. Unfortunately though, the reactivity against the keto group was negligible.

Finally, after multiple injections, we experienced undesirable precipitations for most reagents in the ESI source on the spray shield. As a general rule, the more pronounced the precipitation, the more the reagent tended to polymerize, degrade, and form late eluting products not directed to waste during the first minutes of LC separation. In this context, DCCH, which was hardly soluble in water or organic phase other than dimethylformamide, was the reagent that was most prone to precipitation. Its derivatives were indeed expected to exhibit a very low solubility in aqueous solvents, and we experienced particularly serious, hardly removable source contamination during these experiments. Moreover, we observed a fast deterioration of the LC column signal background and performance using this derivatization agent as well as abundant, long-tailing memory peaks. The solutions of this reagent would have to be strongly diluted or the reagent would have to be removed before analysis to avoid any precipitation in the ESI source or accumulation on the column due to precipitation, which might compromise the limit of detection for the method. In the literature, this reagent is instead used for analysis of lipoxidation products from polyunsaturated fatty acids of higher molecular weight compared to MGO [40], where the chromatographic separation of the reagent by elution within the dead volume is easier to achieve with other chromatographic systems than that used for the small RCC investigated here. In addition, reagent removal by liquid extraction of derivatives from the aqueous sample to nonpolar solvents after incubation time might be a useful approach to improve the situation; however, this would lengthen the sample preparation procedure and manual effort.

#### 2.2.3. Reaction Products of Phenylhydrazines Featuring a Permanent Charge Provide Excellent Solubility but Insufficient Stability

When it comes to reversed phase (RP) LC analysis of aqueous samples, a particular problematic aspect of the hydrazines and their reaction products is the poor solubility in water, which leads to precipitation in the samples, possibly disturbing the injection process or, worse, to precipitation in the separating column. Therefore, we investigated the suitability of permanently charged reagents in analogy to the aniline 4-AEA [24] to improve the solubility of the reagent and any obtained products. Given the results we obtained with our test candidates, including 3-MPH, we also developed synthesis routes leading to the corresponding permanently charged phenylhydrazine (4-AEH), the corresponding 3-substituted phenylhydrazine, and a third hydrazine by additionally introducing bromine as inductive electron-withdrawing substituent in *ortho* position to improve the stability of the molecule (Figure 2b, protocols described in the supplement). These reagents were tested with methylglyoxal.

**Figure 5:**
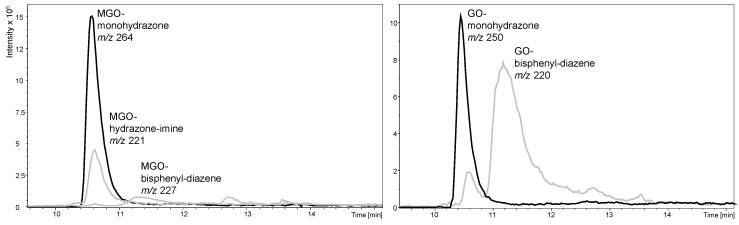
LC-ESI-MS analysis of the reaction mixtures from the derivatization of MGO (**left**) and GO (**right**) with 3-AEH after 1 h incubation time in aqueous ACN. Black: ion current of selective mass traces (extracted ion chromatogram) for the monohydrazone derivatives of MGO, *m/z* 264, and GO, *m/z* 250. Grey: ion current of selective mass traces of the MGO hydrazone-imine and bisphenyldiazene (*m/z* 221 and 227) and GO bisphenyldiazene (*m/z* 220) derivatives.

As expected, precipitate formation did not appear in the reaction mixtures with these reagents featuring a permanent charge. However, unfortunately, the synthesized hydrazines were still very unstable in aqueous solution and produced several oxidation products at later retention times. Thus, the signal response of all synthesized hydrazines was significantly decreased after just a few hours (data not shown), indicating a particularly bad stability of the reagents in aqueous solution. The reaction with MGO appeared to be very fast as the maximum response was observed as early as after 30–45 min incubation, while the derivative with bromine as substituent provided the worst response (data not shown), possibly due to a lower reactivity of the starting material from the inductive electron-withdrawing effect or a worse ionization efficiency of the derivative. Figure 5 illustrates the appearance of the chromatogram of the reagent mixtures with 3-AEH after 1 h incubation time. 

Unfortunately, in addition to the insufficient stability of the reagents, a clear discrimination in reactivity against the keto (MGO) and even the second aldehyde group (GO) obviously led to abundant multiple derivatives, exhibiting a disadvantage of the use of these reagents in the development of multiselective methods, including ketones as target compounds. Finally, though the permanently charged hydrazines produced derivatives with satisfactory responsiveness (e.g., factor 2.5 for the MGO monoderivative of 4-AEH compared to 3-MPH), in agreement with the behavior of the reagents, the stability of these derivatives was also less satisfactory. Thus, the response of the derivatives obtained with 4-AEH continuously decreased to 50% of the original value during 7 h incubation time (for the 3-AEH derivative, the half-life was as short as ~30 min). The highest response of the derivative was always observed with the shortest possible incubation time.

The corresponding anilines featuring a permanent charge [24] were expected to be more stable than the hydrazines. However, we did not obtain any products in the course of 10 h without adding sodium cyanoborohydride as reduction agent for the formed imines to the corresponding amines, which could only then be detected with high sensitivity. As reported, the keto group of MGO hardly reacted, meaning these reagents would only be suitable for reactive carbonyls carrying an aldehyde function.

In conclusion, among the reagents whose derivatives exhibited a high signal response after reaction with MGO, we found 3-MPH and 4-PDA produced the least number and intensity of by-products, which is an important advantage when it comes to the establishment of multitargeted methods. While MGO derivatives were successfully separated from the most abundant by-products of all investigated reagents, derivatives of other carbonyl compounds might coelute and therefore be prone to be suppressed by these abundant signals as was found for the derivative of 3-deoxyglucosone and the 4-PDA dimer at *m/z* 241 (data not shown). In such situations, further method optimization, such as improvement of chromatographic separation or the addition of 2-mercaptoethanol to reduce the reagent dimer formation, may be recommended.

### 2.3. Application of 3-MPH as Derivatizing Agent to Explore the Contamination of Laboratory Water with Methylglyoxal

**Figure 6:**
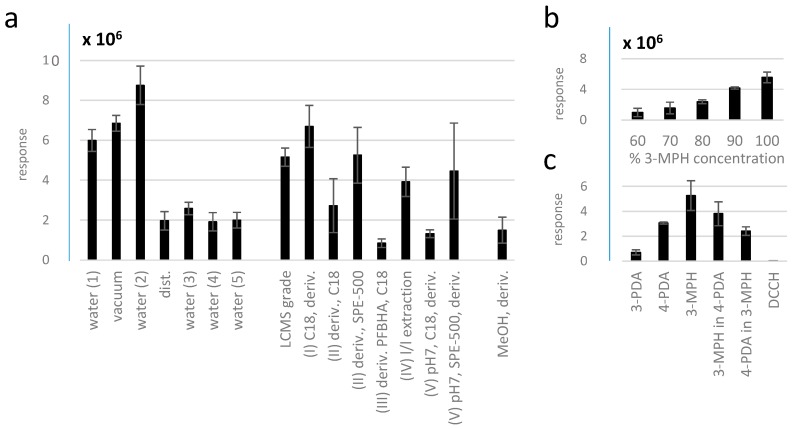
(**a**) Water contamination with MGO analyzed as MGO–dihydrazone after incubation with 3-MPH. Different quality laboratory water and different procedures for purification of water were tested, refer to Section 2.3. (**b**) Response of the MGO–dihydrazone in preparations containing different concentrations of 3-MPH in relation to the original 5 mM solution. (**c**) Response of the corresponding MGO derivatives in blank solutions of four different reagents and response of the dihydrazone after incubation of a 3-MPH solution with 4-PDA and the quinoxaline in a 4-PDA solution with 3-MPH.

Another well known, serious problem in the quantitative analysis of methylglyoxal is the fact that it is hard to produce a clean solvent blank as required, for instance, for LOD determination [41]. As illustrated below using 3-MPH as reagent, even high-quality equipment and several tested cleaning procedures were not able to completely remove methylglyoxal from the blank (Figure 6).

MGO response in methanol equaled the one from distilled water and water purification systems 3, 4, and 5, suggesting that these purification protocols indeed performed the best. However, successful purification by SPE procedures appeared to be much more cumbersome, expensive, and laborious and required a very careful optimization in advance. Thus, we believe the favorable value achieved with the PFBHA (III) protocol could at least partially be a consequence of signal suppression by bulk unreacted PFBHA, which was not satisfactorily removed by SPE. Excess PFBHA is usually removed by l/l extraction after selective protonation of the reagent [16], after which a higher response of blank contamination can be obtained (protocol IV). Moreover, complete elimination of the reagent employed for MGO removal from the solvent used for analysis is indispensable to avoid subsequent competitive reaction with the reagent of choice for determination of MGO in the sample. Using the same reagent for purification of water and analytical derivatization itself [26], on the other hand, may still lead to an overestimation of MGO in the sample in case the residue is not completely removed.

Curiously, we still obtained a response for MGO when injecting 3-MPH in methanol only. This finding prompted us to further test the potential origins of the contamination. Considering that excess reagent is used for derivatization, MGO response in the water blank was expected to stay constant with decreasing reagent concentration until a certain threshold. Instead, we observed an immediate decrease in response with decreasing concentrations of the reagent at all levels (Figure 6b), indicating reagent contamination with the derivative. Reagent contamination was further confirmed by dissolving one reagent in a solution of another after 2 h incubation, i.e., 3-MPH in 4-PDA and 4-PDA in 3-MPH solutions without adding MGO, where derivatives of MGO with both reagents were found (Figure 6c). We conclude that the reagents themselves are a source of blank contamination and require thorough purification and subsequent storage under appropriate conditions, particularly if quantification near or below the concentration of the corresponding contamination is anticipated (5 nM for 3-MPH in our case). In addition, appropriate blank replication is required for quantification above the blank level for which, theoretically, the reagents can be used without prior purification employing blank subtraction. Thus, 3-PDA, 4-PDA, and 3-MPH were all suitable to determine concentrations down to 10 nM MGO (0.1 pmol injected) before blank contamination prevented quantification of lower concentrations. For comparison, typical concentrations of MGO in biological samples are, for example, ~250 nM in whole blood and 170 nM in human plasma [17], 1 nmol/g in U87 cancer cells [22], ~15 µM in wine [42], and >200 nM in urine [26].

### 2.4. Analysis of Carbonyl Reaction Products of Linoleic and Linolenic Acids Oxidation by Cu(II) and Hydrogen Peroxide After Derivatization with 3-MPH

As a proof of concept and to show the applicability of our derivatization agent, we analyzed reaction mixtures after oxidation of linoleic and linolenic acids—two very important native fatty acids that are present, for instance, in human epidermis. Oxidation of these acids is known to produce mainly hydroxylated acids, such as 8,13-dihydroxy-9,11-octadecadienoic and 9,14-dihydroxy-10,12-octadecadienoic acids [43], but oxo-octadecadienoic acids were also observed [44]. Recently, the formation of several carbonyl compounds, such as acrolein and crotonaldehyde as well as glyoxal and methylglyoxal after oxidation of linoleic and linolenic acids, has been reported [45] but no quantitative information has been added. Because lipoxidation mechanisms are a highly interesting topic in biomedical research and because there is already information available to rate the validity of our own results, we selected this model system to show the performance of our method. Firstly, we detected signals that could be produced by approximately 60 possible RCC candidates (although this tentative identification would have to be subjected to future confirmation). Moreover, we were able to quantify methylglyoxal in the reaction mixture (4.6 µM in the linoleic acid and 1.1 µM in the linolenic acid mixtures) and used the average comparison for other test candidates. As an example, we present a small table with ratios and average comparisons of selected potential target compounds (Table 2).

Although an exhaustive evaluation of such an experiment would require prior establishment of a proper multimethod, in particular the assessment of appropriate retention times and MS-MS data for differentiation of derivatives with the same mass but different structure (e.g., methylglyoxal and malondialdehyde), this experiment already shows the capability of the reagent for application in such multitargeted methods to assess the presence and concentration of many carbonyl compounds in a sample in one analytical run. However, compared to our standard samples, we observed not only a significantly enhanced background in the hydrogen peroxide and metal ion (Cu II)-containing solutions of this experiment but also an enhanced formation of the reagent dimer (*m/z* 243) as indicator of oxidative degradation of the reagent. (Note that similar effects were observed for 4-PDA as derivatization agent in a glycation mixture from a glucose solution with Fe II/III; data is not shown). Copper and iron salts are additives with the particular purpose of enhancing the reactivity of lipids and carbohydrates toward oxidative degradation; therefore, not surprisingly, we found that the presence of these Lewis acids may become very problematic in investigations such as ours. Their interaction with the lone electron pairs of the heteroatoms could enhance the reactivity of the reagents and the carbonyls. Thus, in such experiments, the immediate analysis of the derivatized sample would be of particular importance. Our advanced protocol, which changed the solvent directly after derivatization, is expected to decrease the concentration of these metal ions in the reaction mixture and would therefore provide a significant advantage to improve experimental variance.

## 3. Materials and Methods

### 3.1 Materials and Chemicals

Acetonitrile (ACN; ROTISOLV^®^, ≥99.95%, LC-MS Grade), cyclohexane, and formic acid (>98%, p.s.) were purchased from Carl Roth, Karlsruhe, Germany. Bakerbond SPE-500, C18 and empty cartridges (all J.T.Baker, Philipsburg, MT, USA), LiChrosorb C18 bulk material (Merck, Darmstadt, Germany), and water (HiPerSolv^®^ CHROMANORM^®^, LC-MS Grade) were purchased from VWR Chemicals, Darmstadt, Germany. Sodium cyanoborohydride (95%) was purchased from Alfa Aesar, Karlsruhe, Germany. All other chemicals were purchased from Sigma-Aldrich, Taufkirchen, Germany. The experimental details for synthesis and analytical confirmation of the permanently charged anilines and hydrazines, i.e., 2-(4-hydrazineylphenoxy)-*N*,*N*,*N*-trimethylethan-1-aminium (4-AEH), 2-(2-bromo-4-hydrazineylphenoxy)-*N*,*N*,*N*-trimethylethan-1-aminium (3-BrAEH), and 2-(3-hydrazineylphenoxy)-*N*,*N*,*N*-trimethylethan-1-aminium (3-AEH) bromides and the corresponding aminoethoxyanilines (AEA), are given in the supplementary.

All solvents were degassed with Argon 4.6 (Air Liquide, Düsseldorf, Germany). All reagent solutions were prepared in situ, sonicated, and handled under argon in the dark. The pH was adjusted using the pH meter Level 1 (WTW, Weilheim, Germany) with the pH electrode BlueLine 24 pH (Schott Instruments, Mainz, Germany).

MGO contamination was analyzed in water from the following water purification systems: BWT Permaq Pico 10-90 (1, BWT Wassertechnik GmbH, Schriesheim, Germany), Veolia Berkesoft Midi with Berkefeld miniRO 5 (2, Veolia Water Technologies, Celle, Germany), Millipore Milli-Q Integral 5 (3), Millipore Elix 3 with Element A10 (4), and Millipore Direct-Q 3UV (5); the latter three were from Merck, Darmstadt, Germany.

### 3.2. Response and Stability of Methylglyoxal Derivatives

A total of 21 reagents were used for analytical derivatization of methylglyoxal with subsequent LC-MS analysis of the derivatives. The detailed structures of all reagents are illustrated in Figure 2, and all used abbreviations are summarized in Table S2. The selection of the reagents was guided by reported frequent use for carbonyl derivatization and mainly comprised two groups of chemical substances: aromatic amines [41,46] and hydrazines [47]. In our experiments, we included further reagents from these two groups with substituents, allowing a more systematic comparison of signal response featuring electron-withdrawing and electron-donating substituents in different positions to the reacting amino group. 

Below, we describe the protocols used for derivatization as adopted from the literature and optimized if required. Stability of the reagents and the reaction mixtures was assessed over the course of one week.

#### 3.2.1. Reaction with Anilines

Anilines were dissolved in 50 mM ammonium acetate pH 5.7 to prepare mixtures of 2.5 mM reagent and 25 µM MGO [24]. Alternatively, these samples contained 2.5 mM sodium cyanoborohydride as reduction agent. Reagent blanks and reagent mixtures without sodium cyanoborohydride were analyzed by LC-MS after 1, 3, and 10 h incubation time; reagent mixtures with sodium cyanoborohydride were analyzed after 3 h incubation time [24]. Five millimolar 4-aminopyridine and 250 µM MGO in 20% ACN (*v*/*v*) were incubated for 2 h and analyzed in triplicate by LC-MS after appropriate dilution.

#### 3.2.2. Comparison of Phenylenediamines (PDA)

Responsiveness of formed quinoxalines was studied after mixing equal volumes of 16 mM reagent stock solutions in methanol (pH 4) with 3 mM MGO or GO for incubation at 37 °C for 70 minutes in the dark at 550 rpm shaking. Reaction mixtures were diluted and analyzed with flow injection analysis in ESI positive mode.

#### 3.2.3. Reaction with Phenylhydrazines, 4-Hydrazinopyridine, and Methoxyphenylenediamines

Ten millimolar 4-methoxyphenylhydrazine (4-MPH), 3-methoxyphenylhydrazine (3-MPH), phenylhydrazine (PH), 4-hydrazinopyridine (4-HP), 3-methoxyphenylenediamine (3-PDA), and 4-methoxyphenylenediamine (4-PDA), were used to prepare reaction mixtures containing 50 µM MGO in 20% ACN (*v*/*v*) at pH ~4. For the synthesized hydrazines, 50 mM ammonium acetate pH 5.7 was used instead (n = 3) [24]. Reaction vessels were closed firmly and incubated for 2 h shaking in the dark before analysis by LC-MS.

#### 3.2.4. Reaction with the Amplifex™ Keto Reagent, Methoxyamine, and 7-(Diethylamino)coumarin-3-carbohydrazide

The derivatization with the Amplifex™ Keto Reagent Kit (AKR) [33] was carried out according to the manufacturer’s instructions (ABSciex, Framingham, MA, USA). Briefly, 50 µM MGO was incubated with the reagent working solution for 1 h at room temperature.

Ten millimolar aqueous methoxyamine (MOA) and 100 µM MGO were incubated for 2 h at room temperature, diluted, and analyzed in triplicate.

Derivatization with 7-(diethylamino)coumarin-3-carbohydrazide (DCCH) was accomplished in triplicates as described in Reference [40]: 5 µL of a 100 mM stock solution in DMF was mixed with 40 µL 50% ACN and added with 5 µL of a 10 mM MGO solution. The mixture was incubated for 1 h at 37 °C and diluted with 20% ACN (1:20) prior to LC-MS analysis.

#### 3.2.5. Relative Response for Different Types of Reagents

Prior to comparison of responsiveness, we confirmed the dynamic behavior from 10 to 300 µM MGO (n ≥ 3). Based on this investigation, we selected a concentration of 50 µM (0.5 nmol on column) in the final sample as the basis for our comparison (n ≥ 3). This concentration is in the lower dynamic range and not hampered by any blank contamination. We refrained from comparison of responsiveness at high concentrations because the concentration of RCCs in biological and environmental samples is usually rather low.

However, for particularly successful protocols, we additionally estimated the limit of detection (S/N ≥ 3). A factorial dilution series (factor 3) of the derivatization products of MGO with 3-PDA, 4-PDA, 4-MPH, 3-MPH, DCCH, AKR, 3-trimethylammonioethoxyphenylhydrazine (3-AEH), and 3-trimethylammonioethoxyaniline (3-AEA) was used to assess the agreement with the relative response at 50 µM.

#### 3.2.6. Optimized Derivatization Protocol with 3-MPH

Ten millimolar 3-MPH in methanol was mixed with an equal volume of the sample in 50 mM ammonium acetate buffer of pH 4 and incubated for 30 min in the dark. Subsequently, the sample was completely evaporated in a vacuum centrifuge. The sample was dissolved in methanol before LC-ESI-MS analysis. If diluted in an aqueous solvent, dilution was made very shortly before analysis of the sample.

### 3.3. Application of 3-MPH as Derivatization Agent to Explore the Contamination of Laboratory Water with Methylglyoxal 

We determined MGO blank contamination in water from five different water purification systems as outlined under 3.1. In addition, several protocols were assessed for their ability to completely remove methylglyoxal from blank water (HiPerSolv^®^ CHROMANORM^®^, LC-MS Grade from VWR Chemicals, Darmstadt, Germany).

Thus, apart from heating for 20 min at 100 °C under the fume, distillation, and vacuum centrifugation without heating to reduce the solvent volume by ~25% before LC-MS analysis, we also applied several solid phase extraction protocols. For solid phase extractions, cartridges were conditioned with methanol before application: (1) Water was applied to a Bakerbond C18 cartridge and the eluate was subjected to derivatization with 3-MPH. (2) Water was derivatized with 3-MPH and applied to SPE extraction with Bakerbond C18, LiChrosorb C18 and SPE-500. (3) 25 mg *O*-(2,3,4,5,6-pentafluorobenzyl) hydroxylamine (PFBHA) was dissolved in 20 mL water, incubated 1 h at 40 °C, applied to SPE extraction with Bakerbond C18, and subsequently derivatized with 3-MPH. (4) 2.5 mg/mL PFBHA in water was subjected to l/l extraction against equal volumes of cyclohexane. The aqueous phase was subjected to derivatization with 3-MPH. (5) Water was adjusted to pH 7 by ammonia, extracted with Bakerbond C18 and SPE-500 and subsequently derivatized with 3-MPH. 

All purified extracts were subjected to LC-MS analysis (n ≥ 3).

### 3.4. Analysis of Carbonyl Reaction Products of Linoleic and Linolenic Acids Oxidation by Hydrogen Peroxide After Derivatization with 3-MPH

Three replicates of the following samples were prepared in 100 µL water: 500 nmol linoleic or linolenic acids (5 mM, 1.4 mg/mL) and 300 nmol Cu(NO_3_)_2_ (3 mM) were added with 10 µmol hydrogen peroxide (100 mM) and incubated overnight at 37 °C while shaking. Afterwards, samples were derivatized with 3-MPH as described under 3.2.6 and analyzed by LC-ESI-MS as described below.

### 3.5. Instrumental Parameters of LC-ESI-MS

LC-MS analysis was carried out using an HPLC system 1100 series (Agilent, Waldbronn, Germany) equipped with a Gemini C18 reversed-phase column 5 µm, 110 Å, 150 mm × 2 mm (Phenomenex, Aschaffenburg, Germany). For separation of the derivatives obtained with the permanently charged Amplifex™ Keto Reagent (Figure 2a), 3-AEH and 3-AEA, an Accucore aQ LC column (100 × 2.1 mm, 2.6 µm, Thermo Fisher Scientific GmbH) was used. The columns were kept at 40 °C, and 10 µL sample was injected.

The gradients were established with 0.1% formic acid in ACN as Eluent A and in water as Eluent B according to the required performance (Table 3).

The LC system was coupled with a Bruker ESI ion trap mass spectrometer Esquire 3000+ (Bruker Daltonik GmbH, Bremen, Germany) operated in positive ion mode. The source parameters were as follows: nitrogen as nebulizer (55 psi) and dry gas (12 L/min) with a temperature of 365 °C; ICC 20,000, target mass *m/z* 250, scan range *m/z* 50–700.

Flow injection analysis by full scan MS was carried out on the Esquire 3000 Plus using a syringe pump (KD Scientific, Hollisten MA, USA) at a flow rate of 4 µL/min. For this, the nebulizer was set to 11 psi, and the dry gas was set to 5 L/min at 280 °C. Target mass was set to the anticipated *m/z* value and a scan range from *m/z* 50–500.

Bruker Data analysis 4.2 (Bruker Daltonik GmbH, Bremen, Germany), was used for raw data assessment based on peak areas (LC-MS analyses) or peak height (flow injection analysis) of selected mass traces for the derivatives listed in Table S1.

Student’s *t*-test for average comparisons was performed with MS Excel 2013 (Microsoft, Redmond, WA, USA).

## 4. Conclusions

In our survey, the commercially available substances 4-methoxyphenylenediamine followed by 3-methoxyphenylhydrazine were the best performing reagents for derivatization of methylglyoxal in LC-ESI-MS analysis considering handling, analysis time, sensitivity of the derivatives, and stability of the reagent mixture at room temperature. With respect to reactive carbonyl compounds in general, permanently charged anilines might still be a better choice for derivatization of aldehydes with subsequent LC-MS analysis due to the high response and stability of the corresponding *N*-substituted anilines obtained after reductive amination of the formed imines; however, they cannot be employed for ketones. Moreover, the very quick reaction time achieved with 4-PDA and 3-MPH exhibits a great advantage over reagents requiring longer incubation times, especially because MGO can be released or produced de novo from certain sample matrix components during procedural steps and because the remaining metabolic activity in biological samples may result in an overestimation of its concentration in the sample [41]. Finally, apart from stability, sensitivity can also be greatly enhanced when drying the samples and dissolving in a smaller volume of a nonaqueous solvent. Using this improved protocol, we showed the capability of employing 3-MPH for quantification of methylglyoxal during a lipoxidation experiment and demonstrated the potential of this method to analyze different carbonyl compounds simultaneously.

A particularly bothersome fact, however, appeared to be the solvent and reagent contamination with methylglyoxal at very low concentration levels. While contamination of water used as sample solvent could be removed by repeated distillation or high-quality water purification systems, the development of a protocol for satisfactory reagent purification is tedious and requires very careful optimization.

In addition, the stability of the derivatives with trapping reagents still needs very careful consideration, particularly when applying lengthy deproteinization procedures as often required for biological samples or extended trapping times of MGO from environmental samples. Here, an insufficient stability of the derivatives may lead to an underestimation of MGO concentration in the samples. With only intermediate stability of the derivatives, nested online sampling and derivatization [48,49] might be considered when analyzing large sample batches.

## Figures and Tables

**Table 1:** Limit of detection (LOD, S/N > 3) for MGO derivatives of seven different reagents with LC-ESI-MS, estimated from factorial dilution series factor 3 involving concentrations below detection.

	LOD pmol/Injected	Literature Reference Value LOD pmol/Injected
4-PDA	0.01	0.1 with HPLC-FD [26]
3-MPH	0.05	n.a.
3-AEH	0.7	n.a.
3-AEA	1	n.a. ^a^
DCCH	1.5	n.a. ^b^
AKR	2	n.a. ^c^
4-MPH	>50	n.a.

^a^ 0.03 pmol/injected for malondialdehyde derivatized with 4-AEA analyzed with LC-ESI-MS/MS [24]; ^b^ 1 nmol/L 4-hydroxynonenal analyzed with continuous flow nanospray mass spectrometry [32], ^c^ 0.035 fmol testosterone analyzed with LC-ESI-MS/MS [33].

**Table 2:** Average comparison of selected, tentatively identified carbonyl compounds between the reaction mixtures of linoleic and linolenic acids. Values below one represent candidates that were more abundant in linolenic acid mixtures, and those above one represent candidates preferentially formed from linoleic acid.

Tentative Identification	Ratio C18:2/C18:3	*p*-Value *t*-Test
oxobutanal *	11	0.005
oxopentanal *	10	0.056
oxononanoic acid *	1.9	0.035
methylglyoxal	1.7	0.013
glyoxal	0.7	0.031
acrolein *	0.7	0.056
pentenal *	0.6	0.071
malondialdehyde *	0.4	0.015
hexenedial *	0.1	0.005

* tentative assignment by *m/z*.

**Table 3:** Gradients used for HPLC separation of the carbonyl derivatives.

Experiment	Gradient [min]/[%B]	Flow mL/min
3- and 4-MPH, PDAs and MOA	0/80, 5/80, 20/70, 30/30, 35/0, 42/0, 47/80, 54/80	0.6
DCCH, 4-AEH	0/90, 5/90, 15/0, 30/0, 40/90, 45/90	0.5
Amplifex™ (AKR)	0/0, 10/0, 30/50, 40/98, 45/98, 46/0, 56/0	0.3
3-AEH, 3-AEA	0/98, 5/98, 15/0, 20/0, 20.1/98, 25/98	0.3

## Supplementary Materials

The following are available online at http://www.mdpi.com/1420-3049/23/11/2994/s1, Materials and Methods for the synthesis of the permanently charged hydrazines and anilines; Table S1: Selective *m/z* for quantification of the MGO derivatives, Table S2: Abbreviations of the used reagents.
