# Supplementary material for: Derivatization of Methylglyoxal for LC-ESI-MS Analysis—Stability and Relative Sensitivity of Different Derivatives
Source: Molecules. 2018 Nov 16;23(11):2994. doi: 10.3390/molecules23112994 (PMC6278547; doi:10.3390/molecules23112994)
Supplement: Supplementary file 1 [file molecules-23-02994-s001.pdf]

molecules

MDPI

Supplementary

Derivatization of Methylglyoxal for LC-ESI-MS Analysis—Stability and Relative Sensitivity of Different Derivatives

Stefan Fritzsche [1,2], Susan Billig [1,], Robby Rynek [1], Ramarao Abburi [1,3], Elena Tarakhovskaya [1,4], Olga Leuner [1,5], Andrej Frolov [6] and Claudia Birkemeyer [1,*]

[1] Institute of Analytical Chemistry, Faculty of Chemistry and Mineralogy, University of Leipzig, 04103 Leipzig, Germany; stefan.fritzsche@uni-leipzig.de (S.F.), billig@uni-leipzig.de (S.B.), r.rynek@live.de (R.R.), rams.abburi@gmail.com (R.A.), elena.tarakhovskaya@gmail.com (E.T.), leuner@ftz.czu.cz (O.L.);
[2] Institute of Pharmacy, Faculty of Medicine, University of Leipzig, 04103 Leipzig, Germany;
[3] Department of Chemistry, Krishna University, Machilipatnam-521001, Andhra Pradesh, India; rams.abburi@gmail.com
[4] Department of Plant Physiology and Biochemistry, Saint Petersburg State University and Russian Academy of Science Library, Saint Petersburg 199034, Russian Federation;
[5] Faculty of Tropical AgriSciences, Czech University of Life Sciences Prague, 165 00 Praha-Suchdol, Czech Republic;
[6] Department of Biochemistry, St. Petersburg State University, Saint Petersburg 199034, Russian Federation; Andrej.Frolov@ipb-halle.de;
* Correspondence: birkemeyer@chemie.uni-leipzig.de; Tel.: +49-341-973-6092

Synthesis of the permanently charged hydrazines and anilines

1.1. Materials and chemicals

Acetonitrile (ACN; ROTISOLV®, ≥ 99.8%, for preparative HPLC), formic acid (> 98%, p.s.), n-hexane (ROTISOLV®, ≥ 95%, GC Ultra Grade), potassium bromate (p.a.), zinc dust (98%), methanol (ROTISOLV®, ≥ 99.95%, LC-MS Grade) and diethyl ether (ROTIPURAN®, ≥ 99.5%, p.a.) were purchased from Carl Roth, Karlsruhe, Germany. Ethanol (absolute, > 99.7%, HiPerSolv® CHROMANORM®), cyclohexane, 4-hydroxyacetanilide (p.s., ≥ 99.0%), sodium hydrogen carbonate, sodium sulfite (purum, anhydrous), potassium carbonate and hydroxide, ammonia (35%, p.a.), acetic (100%, p.a.) and sulfuric acid (98%, p.a.) were purchased from VWR Chemicals, Darmstadt, Germany. Sodium hydroxide (pellets, 97%, A.C.S. reagent), and hydrobromic acid (purum, p.a., ≥ 48%) were purchased from Sigma-Aldrich, Taufkirchen, Germany. 1,2-Dibromoethane (99%), trimethylamine (33% in ethanol), sodium cyanoborohydride (95%), and 3-hydroxyacetanilide (99%) were purchased from Alfa Aesar, Karlsruhe, Germany. Sodium nitrite (p.a.), methyl ethyl ketone (purissimum, p.a.), sodium bromide (p.a.) and phosphoric acid (85%, purum) were from VEB Laborchemie Apolda, Germany; sodium dithionite (p.a.) from Ferak, Berlin, Germany, ethanol (technical grade, absolute, 1% toluene) from Brüggemann Alcohol, Heilbronn, Germany. D_2O, D_3COD and DMSO-d_6 were kindly provided by Dr. Lothar Hennig, Universität Leipzig. Argon 4.6 from was from Air Liquide, Düsseldorf, Germany.

A Vario EL III (Elementar, Hanau, Germany) was used for elemental analysis. NMR and electrospray – ionization - mass spectrometry ($M+H^+$) were employed for confirmation of all synthesized compounds (not shown). NMR-spectra were acquired on a Mercury 400 plus or Mercury 300 plus (Varian) and calibrated based on the residue signal of D_2O, D_3COD and DMSO-d_6. Mass spectra were acquired after flow injection on an Esquire 3000+ ESI iontrap MS (Bruker Daltonik, Bremen, Germany).

Bromine was produced by reacting potassium bromate with stoichiometric amounts of concentrated hydrobromic acid and then dried by stirring with concentrated sulfuric acid.

Anhydrous hydrogen bromide was obtained after adding sodium bromide under slight warming to concentrated phosphoric acid dehydrated by heating with a hot air gun.

Potassium carbonate was dried at 200 °C for at least 2 h before use.

1.2. Synthesis of 3-bromo-4-hydroxyacetanilide

7.6 g (50.0 mmol, 1.0 eq) 4-hydroxyacetanilide was suspended in 80 mL glacial acetic acid. 2.6 mL (8.1 g, 50.7 mmol, 1.0 eq) bromine in 10 mL glacial acetic acid was added while stirring. After 2 days, the reaction mixture was cooled to 10 °C, the precipitate was collected and washed with 10 mL glacial acetic acid. After vacuum drying over potassium hydroxide, 10.1 g (88 %) of the brominated phenol was obtained as a light beige solid. ^{1}H-NMR (300 MHz, D_3COD) δ = 7.74 (d, *J* = 2.6 Hz, 1 H), 7.26 (dd, *J* = 2.6 Hz, 8.8 Hz, 1 H), 6.84 (d, *J* = 8.8 Hz, 1 H), 2.1 (s, 3 H) ppm [1].

1.3. Synthesis of 2-bromoethoxyacetanilides [2]

The corresponding phenol (1.0 eq), potassium carbonate (2.5 eq) and 1,2-dibromomethane (5.0 eq) were heated 9-11 h under reflux in acetonitrile (2.5 mL/mmol phenol).

4-(2-bromoethoxy)-acetanilide: The reaction mixture (20 mmol phenol) was poured into 50 mL water containing 100 g ice, the resulting precipitate was filtered off and washed with 50 mL water. The dry solid was dissolved in 10 mL hot ethanol, filtered hot through a fluted filter and washed with 5 mL hot ethanol. The filtrate was heated to boil and mixed with 10 mL of water. After cooling down to room temperature, cooling continued in an ice bath. The precipitate was filtered and washed with 10 mL water and dried under vacuum. A light beige solid were obtained. ^{1}H-NMR (300 MHz, D_3COD) δ = 7.43 (m, 2 H), 6.90 (m, 2 H), 4.27 (t, *J* = 5.9 Hz, 2 H), 3.67 (t, *J* = 5.9 Hz, 2 H), 2.09 (s, 3 H) ppm [3].

3-(2-bromoethoxy)-acetanilide: Reaction mixture (40 mmol phenol) was poured into 300 mL water containing 100 g ice, the resulting precipitate was filtered and dried. The filtrate was reduced to a volume of 100 mL under reduced pressure. The resulting precipitate was filtered and dried. The combined solids were dissolved in 15 mL hot ethanol and the resulting suspension was filtered hot through a fluted filter. After adding 15 mL water and 15 mL ethanol, the filtrate was heated to boil. After cooling in an ice bath, the precipitate was filtered and dried under vacuum. 4.7 g (46 %) of a light beige solid was obtained. ^{1}H-NMR (300 MHz, D_3COD) δ = 7.29 (m, 1 H), 7.20 (m, 1 H), 7.07 (m, 1 H), 6.68 (m, 1 H), 4.28 (t, *J* = 5.9 Hz, 2 H), 3.68 (t, *J* = 5.9 Hz, 2 H), 2.11 (s, 3 H) ppm.

3-bromo-4-(2-bromoethoxy)-acetanilide: The solvent of the reaction mixture (30 mmol phenol) was largely removed on a rotary evaporator and 150 mL of water was added to the residue, vacuum-dried to about 75 mL, and the resulting solids were filtered and washed with 50 mL of water. The solid was dissolved in 40 mL hot ethanol, filtered hot through a fluted filter and washed with 10 mL hot ethanol. After cooling to about 10 °C, the light beige solid precipitate was filtered and washed with 20 mL 50 % ethanol and vacuum-dried. ^{1}H-NMR (300 MHz, D_3COD) δ = 7.84 (d, *J* = 2.6 Hz, 1 H), 7.42 (dd, *J* = 2.6 Hz, 8.8 Hz, 1 H), 6.99 (d, *J* = 8.8 Hz, 1 H), 4.33 (t, *J* = 5.9 Hz, 2 H), 3.70 (t, *J* = 5.9 Hz, 2 H), 2.10 (s, 3 H) ppm.

1.4. Synthesis of quarternary ammonium salts [4]:

2-bromoethoxyacetanilide (1 eq), ethanolic trimethylamine solution (33 %, 4.2 M, 10 eq) and methyl ethyl ketone were stirred at room temperature for 4 days. The precipitate was washed with methyl ethyl ketone. After drying under vacuum, the ammonium salts were obtained as white solids. 2-(4-acetamidophenoxy)-ethyltrimethylammonium bromide: ^{1}H-NMR (400 MHz, D_2O) δ = 7.38 (m, 2 H), 7.06 (m, 2 H), 4.53 (m, 2 H), 3.84 (m, 2 H), 3.28 (s, 9 H), 2.17 (s, 3 H) ppm [29]. 2-(3-acetamidophenoxy)-ethyltrimethylammonium bromide: ^{1}H-NMR (300 MHz, D_2O) δ = 7.40 (t, *J* = 8.2 Hz, 1 H), 7.22 (m, 1 H), 7.04 (m, 1 H), 6.90 (m, 1 H), 4.54 (m, 2 H), 3.84 (m, 2 H), 3.28 (s, 9 H), 2.18 (s, 3 H) ppm. 2-(2-bromo-4-acetamidophenoxy)-ethyltrimethylammonium bromide: The product was further purified by dissolving in the minimum amount of water, filtration and precipitation by adding a mixture of ethanol/diethyl ether/methyl ethyl ketone (*v*/*v*/*v* = 9/2/2) at 10 °C. The resulting

solid was collected by filtration, washed twice with a small volume of methyl ethyl ketone and dried *in vacuo*. Yield: 3.0 g (76 %), ^{1}H-NMR (300 MHz, D_2O) δ = 7.61 (d, *J* = 2.3 Hz, 1 H), 7.28 (dd, J = 2.3 Hz, 8.8 Hz, 1 H), 6.98 (d, *J* = 8.8 Hz, 1 H), 4.48 (m, 2 H), 3.88 (m, 2 H), 2.14 (s, 3 H) ppm.

1.5. Synthesis of aniline hydrobromides [4]:

The corresponding acetanilide (1 eq) was heated 30 min with 5 M HBr (10 eq) under reflux. Products were obtained as white solids.

2-(4-Aminophenoxy)-ethyltrimethylammonium bromide hydrobromide: Excess hydrobromic acid was removed until a total of 10 mL distillate was obtained. After cooling, 2 mL ethanol and 4 mL ether were added. The resulting precipitate was filtered, washed with a mixture of 1 mL ethanol and 3 mL ether and dried. ^{1}H-NMR (400 MHz, D_2O) δ = 7.40 (m, 2 H), 7.17 (m, 2 H), 4.58 (m, 2 H), 3.88 (m, 2 H), 3.29 (s, 9 H) ppm [4].

2-(3-aminophenoxy)-ethyltrimethylammonium bromide hydrobromide: After cooling, the solution was filtered through glass wool and excess hydrobromic acid was removed till 21.5 mL distillate was obtained. 3 mL ethanol and 5 mL ether were added to the cooled residue. The resulting precipitate was filtered, washed with 10 and 5 mL ether and dried under vacuum. ^{1}H-NMR (300 MHz, D_2O) δ = 7.54 (t, *J* = 8.2 Hz, 1 H), 7.17 (m, 1 H), 7.08 (m, 2 H), 4.60 (m, 2 H), 3.89 (m, 2 H), 3.30 (s, 9 H) ppm.

2-(2-bromo-4-aminophenoxy)-ethyltrimethylammoniumbromide hydrobromide: After cooling, the solution was filtered through glass wool and reduced under vacuum to 3 mL. 3 mL ethanol and 15 mL ether were added to the residue. The resulting precipitate was filtered, washed twice each time with 10 mL ether and dried under vacuum. ^{1}H-NMR (300 MHz, D_2O) δ = 7.71 (m), 7.42 (m), 7.21 (m), 4.62 (m, 2 H), 3.94 (m, 2 H), 3.34 (s, 9 H) ppm.

1.6. Synthesis of the hydrazine hydrobromides

360 mg (1 mmol, 1 eq) 4-substituted aniline hydrobromide was dissolved in 500 μL water and 380 μL hydrobromic acid (5 M, 1.9 mmol, 1.9 eq). A cooled solution of 75.3 mg (1.1 mmol, 1.1 eq) sodium nitrite in 300 μL water was added dropwise while stirring and cooling in an ice bath. The color of the solution changed from violet to dark red. The obtained diazonium salt solution was added dropwise to a solution of 276 mg (2.2 mmol, 2.2 eq) sodium sulfite and 61 mg (1.5 mmol, 1.5 eq) sodium hydroxide in 2 mL water in an ice bath. After 15 min, the ice bath was removed and 300 mg precipitate was obtained as a canary yellow solid after filtering and drying under vacuum.

534 mg (1.5 mmol, 1 eq) 3-substituted aniline hydrobromide was dissolved in 450 μL water and 450 μL (5 M, 2.3 mmol, 1.5 eq) hydrobromic acid. To this, a cooled solution of 111 mg (1.6 mmol, 1.1 eq) sodium nitrite in 600 μL water was added dropwise within 10 min while stirring and cooling in an ice bath. The color of the solution changed from orange-red to yellow-brown. The resulting diazonium salt solution was added dropwise to 380 mg (3 mmol, 2.0 eq) sodium sulfite in 3 mL water in 10 min. After reaction, the solution remained overnight in the ice bath, the resulting precipitate was filtered and dried under vacuum to obtain 250 mg of an orange solid.

435 mg (1.0 mmol, 1 eq) brominated aniline hydrobromide was dissolved in 100 μL water and 300 μL hydrobromic acid (5 M, 1.5 mmol, 1.5 eq). A cooled solution of 74.6 mg (1.1 mmol, 1.1 eq) sodium nitrite in 200 μL water was added within 5 min while stirring and cooling in an ice bath. The color of the solution changed from wine red to brown. The obtained diazonium salt solution was added dropwise to a solution of 280 mg (2.2 mmol, 2.2 eq) sodium sulfite and 140 mg (1.0 mmol, 1.0 eq) potassium carbonate in 1.5 mL water within 10 min and incubated overnight in the ice bath. After filtration and drying under vacuum, 296 mg of a yellow solid was obtained.

All diazosulfonates were used without further purification for the subsequent reduction.

150 mg (0.5 eq) of the crude 4-substituted diazosulfonate was dissolved in 5 mL hot water. 79.4 mg (1.2 mmol, 1.2 eq) zinc dust was added in portions. The suspension was heated to reflux for 10 min, the colourless solution was then filtered hot through cotton wool into a flask filled with argon and the residue was washed with 4 mL hot water. After removing the solvent, 148 mg of a pale yellow, viscous residue was obtained.

222 mg (0.88 eq) of the crude 3-substituted diazosulfonate was dissolved in 12 mL hot water and 88 mg (1.35 mmol, 0.9 eq) zinc dust was added in portions. The suspension was heated to reflux for 5 min, the colourless solution was filtered hot through glass wool into a flask filled with argon and the residue was washed with 2 ml hot water. After removing the solvent, 232 mg of an orange-yellow, viscous residue was obtained.

233 mg (0.8 eq) of the crude brominated diazosulfonate was dissolved in 2 mL hot water and 100 mg (1.5 mmol, 1.5 eq) zinc dust was added in portions. After addition of 200 µL glacial acetic acid and slight warming, the colorless solution was filtered hot through glass wool into a flask filled with argon. The residue was washed with 1 mL hot water. After removing the solvent, 344 mg of a white residue was obtained.

2-(4-hydrazinophenoxy)ethyltrimethylammoniumbromide hydrobromide: The residue from the previous synthesis was re-dissolved in 5 mL cold ethanol, mixed with 140 µL 48 % hydrobromic acid and sonicated. Under slight warming and the addition of further hydrobromic acid (390 µL, 48 %), a light yellow solution was obtained. After filtration through cotton wool, 390 µL hydrogen bromide (48 %) and 5 mL diethyl ether were added stepwise to the clear solution while cooling in an ice bath. The precipitate was filtered and washed with 5 mL diethyl ether. After drying under vacuum, 71.0 mg (39 % over the 3 steps) of a dusky-pink colored solid was obtained. ^{1}H-NMR (400 MHz, D_2O) δ = 7.13 (m, 4 H), 4.54 (m, 2 H), 3.85 (m, 2 H), 3.28 (s, 9 H) ppm.

2-(3-hydrazinophenoxy)ethyltrimethylammoniumbromide hydrobromide: The residue from the previous synthesis stage was redissolved in 5 mL cold ethanol, mixed with 220 µL 48 % hydrobromic acid and sonicated. Under slight warming, 620 µL hydrobromic acid (48 %) was added during sonication to obtain an orange solution. After filtration through glass wool into an argon-filled flask, the clear solution was mixed stepwise with a total of 5.5 mL diethyl ether while cooling in an ice bath. The red, viscous precipitate was filtered and washed with 5 mL diethyl ether. After drying under vacuum, 81 mg (16 %, 3 steps) of a pink solid was obtained. ^{1}H-NMR (300 MHz, D_2O) δ = 7.41 (t, *J* = 8.2 Hz, 1 H), 6.82 (m, 1 H), 6.73 (m, 2 H), 4.56 (m, 2 H), 3.86 (m, 2 H), 3.28 (s, 9 H) ppm.

2-(2-bromo-4-hydrazinophenoxy)ethyltrimethylammonium bromide hydrobromide: 2.6 g anhydrous hydrogen bromide was dissolved in 10 mL (7.8 g) absolute ethanol employing anhydrous conditions and ice cooling. The residue from the previous synthesis stage was dissolved in a total of 4.5 mL ethanol and 2.5 mL ethanolic hydrogen bromide solution, treated in an ultrasonic bath and heated to reflux for 5 minutes employing anhydrous conditions. After filtration through glass wool into a flask filled with argon, the clear solution was mixed stepwise with a total of 20 mL diethyl ether while cooling on ice. The resulting white precipitate was filtered and washed three times each with 5 mL diethyl ether. After drying under vacuum, 69 mg (15 %, 3 steps) of a white solid was obtained. ^{1}H-NMR (300 MHz, D_2O) δ = 7.41 (m), 7.14 (m), 4.59 (m, 2 H), 3.92 (m, 2 H), 3.33 (s, 9 H) ppm.

Selective *m*/*z* for quantification of the MGO derivatives

Table S1. selective *m*/*z* used for peak integration to assess the relative responses of derivatives from 19 reagents.

**Reagent.**	***m*/*z* MGO derivative**	**Reagent**	***m*/*z* MGO derivative**
4,5-PDA	189	3- and 4-MPH*	**313** / 296
4-PDA	175	PH*	283
3-PDA	175	3- and 4-AEH (perm. charged hydrazine)	**264** / 228
PDA	145	4-HP*	165

4Cl	179		
4F	163	MOA*	131
4NO	190	Amplifex Keto Reagent (AKR)	151
3NO	190	DCCH*	**330** / 587
		3- and 4-AEA (perm. charged aniline)	251

*sum of both isomers

Table S2. Abbreviations of the used reagents.

		**Phenylenediamines**
1	4,5-PDA	4,5-methylenedioxy-*o*-phenylenediamine (dihydrochloride)
2	4-PDA	4-methoxy-*o*-phenylenediamine (dihydrochloride)
3	3-PDA	3-methoxy-*o*-phenylenediamine
4	PDA	*o*-phenylenediamine
5	4F	4-fluoro-*o*-phenylenediamine
6	4Cl	4-chloro-*o*-phenylenediamine
7	3NO	3-nitro-*o*-phenylenediamine
8	4NO	4-nitro-*o*-phenylenediamine
		**Phenylhydrazines (PH)**
9	4-MPH	4-methoxyphenylhydrazine (hydrochloride)
10	3-MPH	3-methoxyphenylhydrazine (hydrochloride)
11	PH	phenylhydrazine
		Aminoethoxyphenylhydrazines (AEH)
12	4-AEH	2-(4-(2-(trimethylammonio)ethoxy)phenyl)hydrazine
13	3-BrAEH	2-(3-bromo-4-(2-(trimethylammonio)-ethoxy)phenyl)hydrazine
14	3-AEH	2-(3-(2-(trimethylammonio) ethoxy)phenyl)hydrazine
15	DCCH	7-(diethylamino)-coumarin-3-carbohydrazide
16	4-HP	4-hydrazinopyridine hydrochloride
		**Aminoethoxyanilines (AEA)**
17	4-APC	4-(2-(trimethylammonio)ethoxy)benzeneaminium bromide
18	3-AEA	3-(2-(trimethylammonio)ethoxy)benzeneaminium bromide
19	4-AP	4-aminopyridine
		**Hydroxylamines**
20	MOA	methoxyamine (hydrochloride)
21	AKR	3-(aminooxy)-*N*,*N*,*N*-trimethylpropan-1-aminium

References

1. Schmidt B.; Berger R.; Hölter F. Functionalized alkoxy arene diazonium salts from paracetamol. *Org. Biomol. Chem.* **2010**, *8*, 1406–1414.
2. am Ende C. W.; Fish B. A.; Green M. E.; Johnson D. S.; Mullins P. B.; O'Donnell C. J.; Pettersson M. Y.; Stiff C. M.; Subramanyam C.; Tran T. P.; et. al. Novel bicyclic pyridinones, World Intellectual Property Organization WO 2012/131539 A1, 31. March 2011.
3. Murakami H.; Kawabuchi A.; Matsumoto R.; Ido T.; Nakashima N.; A Multi- Mode-Driven Molecular Shuttle: Photochemically and Thermally Reactive Azobenzene Rotaxanes, *J. Am. Chem. Soc.* **2005,** *127,* 15891–15899.
4. Eggink M.; Charret S.; Wijtmans M.; Lingeman H.; Kool J.; Niessen W.M.A.; Irth H. Development of an on-line weak-cation exchange liquid chromatography-tandem mass spectrometric method for screening aldehyde products in biological matrices, *J. Chrom. B* **2009,** *877*, 3937–3945.
